# Genome-Wide Characterization of Endogenous Retroviruses in *Bombyx mori* Reveals the Relatives and Activity of *env* Genes

**DOI:** 10.3389/fmicb.2018.01732

**Published:** 2018-08-03

**Authors:** Min Feng, Xiong Wang, Feifei Ren, Nan Zhang, Yaohong Zhou, Jingchen Sun

**Affiliations:** Guangdong Provincial Key Laboratory of Agro-Animal Genomics and Molecular Breeding, College of Animal Science, South China Agricultural University, Guangzhou, China

**Keywords:** endogenous retroviruses, *Bombyx mori*, NPVs, env, genome

## Abstract

Endogenous retroviruses (ERVs) are retroviral sequences that remain fixed in the host genome, where they could play an important role. Some ERVs have been identified in insects and proven to have infectious properties. However, no information is available regarding *Bombyx mori* ERVs (BmERVs) to date. Here, we systematically identified 256 potential BmERVs in the silkworm genome *via* a whole-genome approach. BmERVs were relatively evenly distributed across each of the chromosomes and accounted for about 25% of the silkworm genome. All BmERVs were classified as young ERVs, with insertion times estimated to be less than 10 million years. Seven BmERVs possessing the *env* genes were identified. With the exception of the *Orf133 Helicoverpa armigera* nuclear polyhedrosis virus, the *env* sequences of BmERVs were distantly related to genes encoding F (Fa and Fb) and GP64 proteins from Group I and Group II NPVs. In addition, only the amino acid sequence of the BmERV-21 envelope protein shared a similar putative furin-like cleavage site and fusion peptide with Group II baculoviruses. All of the *env* genes in the seven BmERVs were verified to exist in the genome and be expressed in the midgut and fat bodies, which suggest that BmERVs might play an important role in the host biology.

## Introduction

Endogenous retroviruses (ERVs) are remnants of ancient retroviral sequences that were once integrated into a host germ line and have remain fixed in the host genome for generations. Their genomic structure is composed of a central part with one to three major genes (*gag*, *pol*, and *env*) flanked by two long terminal repeats (LTRs) (Misseri et al., 2004). They could show infectious properties when possessing the *env* gene (Teysset et al., 1998). ERVs have been identified in the genomes of many vertebrate and invertebrate species (Terzian et al., 2001; Garcia-Etxebarria and Jugo, 2010, 2016; Bustamante Rivera et al., 2017). Due to structural similarity, ERVs can also be considered to be LTR retrotransposons (Havecker et al., 2004). However, the International Committee on Taxonomy of Viruses includes vertebrate ERVs in the family *Retroviridae*, while insect ERVs (IERVs) belong to the family *Metaviridae* (Fablet, 2014).

Classical vertebrate ERVs, such as ERV3 (human) (Bustamante Rivera et al., 2017), and ev21 and EAV-HP (chicken) (Wang et al., 2013; Feng et al., 2016), might play an important role in their hosts. Thus, there is continued interest in ERVs for the roles they could play in normal host biology and diseases, as well as the risks they pose in xenotransplantation (Chuong et al., 2016; Niu et al., 2017). Compared with vertebrate ERVs, IERVs have also been described for a long time but have received less attention. IERVs that have been identified include *gypsy*, *17-6*, *297*, *ZAM*, *Idefix*, *nomad*, and *tirant* in *Drosophila melanogaster*; *Tv1* in *Drosophila virilis*; *tom* in *Drosophila ananassae*; *TED* in *Trichoplusia ni*; and yoyo in *Ceratitis capitata* (Terzian et al., 2001). Of these, both *gypsy* and *ZAM* have been found to have infectious properties (Kim et al., 1994; Leblanc et al., 2000). In addition, maternal transmission of *gypsy* may be influenced in the presence of the endosymbiotic bacterium *Wolbachia* (Touret et al., 2014). However, the role of IERVs in the host’s biological processes remains an interesting and open question.

Despite the prevalence of ERVs in insect genomes, to date, ERVs in silkworm have not been exploited. It has been found that lepidopteran insects diversified during the Early Cretaceous (Misof et al., 2014). The domesticated silkworm, *Bombyx mori*, is a lepidopteron model insect of economic importance. Initially, only several LTR retrotransposon elements were identified in *B. mori* (Abe et al., 2001), but it was later found that retrotransposable elements are the main structural component of the W chromosome (Abe et al., 2005). With the release of the silkworm genome sequence (Xia et al., 2004), LTR retrotransposons of domesticated silkworm were then systematically screened (Jin-Shan et al., 2005). However, thus far, *B. mori* ERVs (BmERVs) have not been thoroughly identified in the whole-genome sequence.

Previous research has reported that IERVs could become infectious agents via acquiring envelope-like gene from a class of insect baculoviruses (Malik et al., 2000). Nuclear polyhedron viruses (NPVs), as members of the Baculoviridae, have been mainly divided into Group I and Group II according to their different envelope gene (Zanotto et al., 1993). In the family Baculoviridae, two different envelope proteins named GP64 and F have been identified. NPVs in Group I use GP64 as their budded virus fusion protein, whereas NPVs in Group II lack GP64 and utilize F protein (Pearson and Rohrmann, 2002). The homologs of the F protein in Group I baculoviruses and F protein in Group II baculoviruses are, respectively, called Fb and Fa (Pearson and Rohrmann, 2002). However, the relationship of envelope proteins between BmERV and NPVs is still unclear.

In the present study, we took advantage of the newest silkworm genome sequence (from SilkDB V2.0) to perform a comprehensive mining and analysis of ERVs in *B. mori*. To our knowledge, this is the first systematic screening of ERVs in insect. The aim of this study was to carry out a detailed analysis of the BmERVs, extending our knowledge of these elements in invertebrates.

## Materials and Methods

### Identification and Annotation of BmERVs

**FIGURE 1:**
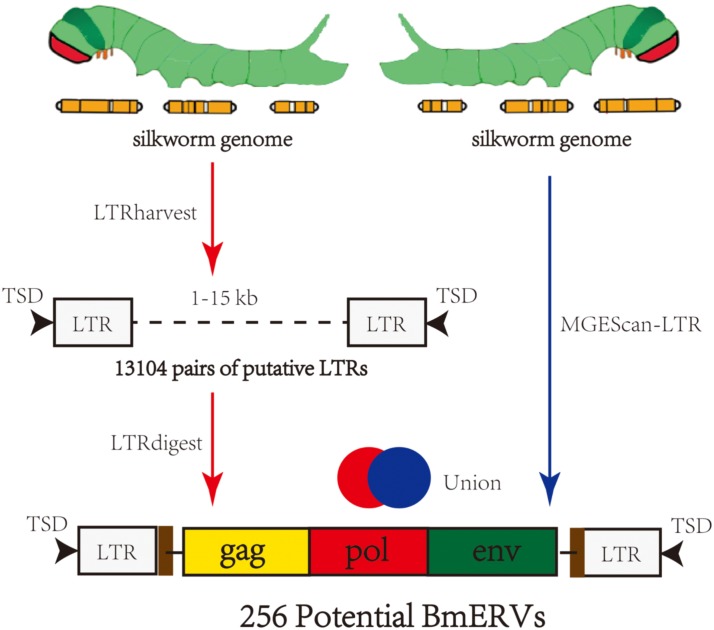
Summary of the strategy used to identify BmERVs. A total of 13,104 pairs of putative LTRs were identified by LTRharvest, and all of them were annotated using LTRdigest. A second pipeline, MGEScan-LTR, was employed to identify BmERVs with the default parameters. Each candidate containing at least three of the five canonical retroviral protein domains (Gag, PR, RT, IN, and RH) was extracted from the results of LTRharvest and MGEScan-LTR as potential BmERVs. Finally, we identified 256 potential BmERVs by merging the ERV candidates identified by the independent pipelines.

The silkworm genome sequence was downloaded from SilkDB V2.0^1^ and used as the input for BmERV identification with two pipelines (**Figure 1**). In the first pipeline, BmERVs were detected in the silkworm genome using the software LTRharvest (GenomeTools1.5.7) (Ellinghaus et al., 2008) and LTRdigest (Steinbiss et al., 2009). Pairs of putative LTRs that were separated by 1–15 kb and flanked by target site duplications were screened by LTRharvest. The threshold of LTR nucleotide similarity used in LTRharvest was greater than 80%; other parameters were set to their defaults. Internal sequence retroviral features of ERV candidates, including protein domains, polypurine tracts, and primer-binding sites, were predicted using LTRdigest with default parameters. A total of 314 retroviral protein domain profiles used for putative ERV domain annotation were downloaded from the Gypsy database.^2^ A second pipeline, MGEScan-LTR, was also employed to identify BmERVs using the default parameters. Each candidate containing at least three of the five canonical retroviral protein domains [Gag, PR, reverse transcriptase (RT), IN, and RH] was retained in the results from LTRharvest and MGEScan-LTR. Finally, the ERV candidates identified by the two pipelines were merged as the BmERV library.

### BmERV Classification and Phylogenetic Analysis

Sequences of the RT domain from BmERVs and known exogenous retroviruses and ERVs (**Supplementary Table S1**) were used for multiple alignment using MUSCLE (v3.8.31) (Edgar, 2004). A neighbor-joining phylogeny was built from the RT domain alignment using MEGA6 with 1,000 bootstrap replicates. The putative families of BmERVs were defined based on their support in phylogenetic trees.

### Insertion Time of BmERVs

When the host neutral substitution rate is known, the integration age of an individual ERV can be estimated by measuring the pairwise distance between LTR sequences. To estimate the insertion time of BmERVs, we used the LTR divergence calculated as *K*/2*r* (Zhuo et al., 2013). *K* is the pairwise evolutionary distance of LTR pairs and *r* is a substitution rate using the *Drosophila* neutral rate of 15.6 × 10^-9^ substitutions per year (Xia et al., 2004).

### RepeatMasker Analysis

Repetitive elements nested within the BmERV library were removed using Repbase (version 17.11) (Bao et al., 2015). The new BmERV library was subsequently used to run RepeatMasker^3^ on the silkworm genome using the sensitive Crossmatch alignment program with the default parameters.

### Analysis of BmERV *env* Genes

BmERVs possessing an open reading frame (ORF) coding for an *env*-like genes, including BmERV-21, BmERV-83, BmERV-94, BmERV-137, BmERV-143, BmERV-145, and BmERV-228, were selected from the BmERV library. The fusogenic region of the BmERV envelope glycoprotein was analyzed using MegAlign (DNASTAR Lasergene.v7.1) and Weblogo.^4^ Several known IERVs including *D. melanogaste*r ZAM virus (ZAM), *D. melanogaster* Idefix virus (Idefix), and *D. melanogaster* Gypsy virus (DmeGypV) were used as references. In addition, sequence alignments were compared between BmERV *env* genes and genes that code for envelope proteins (F and GP64) in Group I and Group II nuclear polyhedron viruses (NPVs) using MegAlign and MEGA6. The GenBank accession numbers of NPV strains are summarized as follows. Group I NPVs: *Autographa californica* nuclear polyhedrosis virus (AcMNPV) cloneC6 (L22858.1), AcMNPV E2 (KM667940.1), *Orgyia pseudotsugata* multinucleocapsid nuclear polyhedrosis virus (U75930.2), and *Bombyx mori* nuclear polyhedrosis virus (BmNPV) (strains Brazilian: KJ186100.1, Guangxi: JQ991011.1, India: JQ991010.1, JapanH4: LC150780.1, Korea C1:KF306215.1, Suzhou Cubic: JQ991009.1, United States T3: KF306215.1, Zhengjiang: JQ991008.1); Group II NPVs: *Lymantria dispar* multiple nucleopolyhedrovirus (LdMNPV)-RR01(KX618634.1), LdMNPV-Ab-a624 (KT626572.1), *Spodoptera exigua* multiple nucleopolyhedrovirus (SeMNPV) HT-SeG25(HG425347.2), SeMNPV VT-SEOX4 (HG425345.1), and *Helicoverpa armigera* nuclear polyhedrosis virus (HaSNPV) (NC_003094.2).

### PCR and RT-PCR Amplification of *env* Genes in *Bombyx mori*

To verify the BmERVs identified by bioinformatics, the *env* genes of BmERVs were amplified with specific primers (**Supplementary Table S2**) using DNA template. RT-PCR was employed to detect the transcription of BmERV *env* genes by using the same primers with cDNA template. DNA and RNA samples were extracted from the fat bodies and midgut of the silkworm at the 5th-instar stage. Samples from five individuals were pooled in equal amounts. cDNA synthesis was performed with a PrimeScript RT Reagent Kit with gDNA Eraser (Takara, Japan) according to the manufacturer’s protocol. In this kit, the first step is to eliminate the genomic DNA using gDNA Eraser.

## Results

### *De Novo* Detection of BmERVs in the Silkworm Genome

Two different pipelines were employed for mining BmERVs in the silkworm genome (**Figure 1**). First, BmERVs were detected by LTRharvest and annotated with LTRdigest. A total of 13,104 ERV candidates with predicted LTR pairs in the silkworm genome were identified with LTRharvest. These candidates were filtered down to 175 ERV candidates by LTRdigest. Additionally, MGEScan revealed 230 ERV candidates. After merging the ERV candidates identified by the two pipelines, we identified a total of 256 BmERVs in the silkworm genome (**Figure 1**). The full -lengths of these 256 BmERVs varied from 1,912 to 20,275 bp, and their LTR lengths ranged from 101 to 687 bp (details of the 256 BmERVs are shown in **Supplementary Data Sheet S1**). Among the detected BmERVs, the most common structures were LTR-*gag-pro-pol*-LTR and LTR-*pol*-LTR (**Table 1**). However, only seven BmERVs contained the *env* gene and three BmERVs contained the complete structure of an insect retrovirus, but none of these had an entire intact sequence (**Table 1** and **Supplementary Figure S1**). This result suggested that entire functional BmERVs might not exist in *B. mori.*

**Table 1:** Structure of BmERVs.

BmERV structure	Number
LTR-*gag*-*pro*-*pol*-LTR	105
LTR-*gag* -*pol*-LTR	41
LTR-*pro*-*pol*-LTR	29
LTR-*pol*-LTR	74
LTR-*pro*-*pol*-*env*-LTR	3
LTR-*pol*-*env*-LTR	1
LTR-*gag*-*pro*-*pol*-*env*-LTR	3


### Phylogenetic Analysis and Classification of BmERVs

**FIGURE 2:**
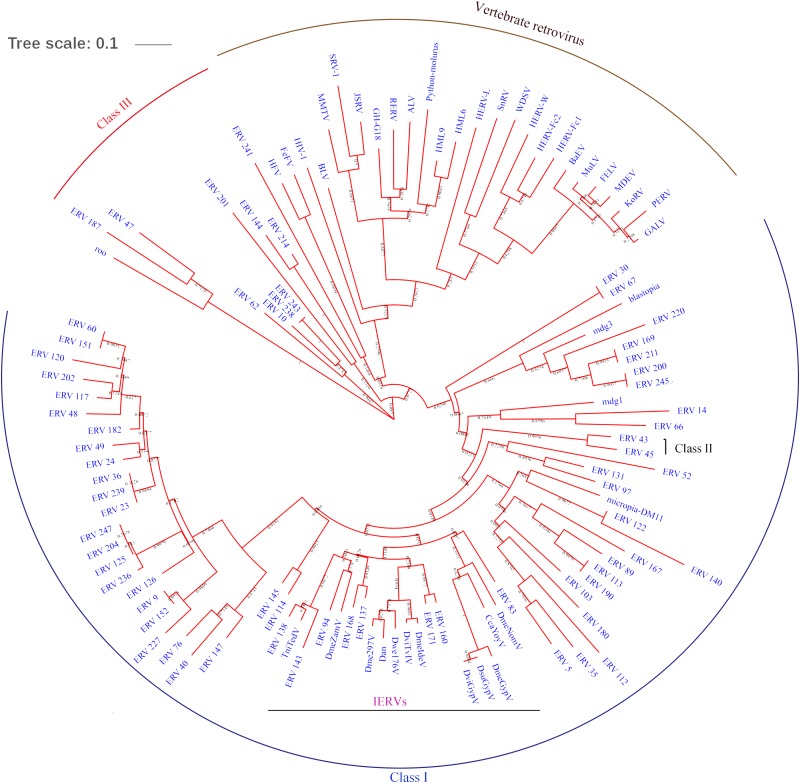
RT region-based phylogenetic tree of BmERVs. A total of 68 BmERVs possessing a RT domain were included. Other retrovirus used in this analysis included vertebrate retrovirus, insect LTR-retrotransposons, and IERVs (**Supplementary Table S1**). Topology was based on the neighbor-joining method with 1,000 bootstraps.

To classify the detected BmERVs, the RT domain sequences of BmERVs, vertebrate retroviruses, and IERVs were used to build a multiple alignment and compute phylogenetic trees using the neighbor-joining method implemented in MEGA 6. Of the 256 BmERVs identified in this sutdy, 68 BmERVs had a RT domain conserved enough to be aligned confidently for phylogenetic analysis. According to the phylogenetic tree analysis, BmERVs were classed into three groups and nine families (**Figure 2** and **Supplementary Data Sheet S2**). Most of the BmERVs included in the phylogenetic analysis belonged to the class I group and were classified into four families (**Supplementary Data Sheet S2**). Additionally, 176 BmERVs were marked as unclassified members (**Supplementary Data Sheet S2**). On the whole, class I and class II BmERVs were related to IERVs from errantivirus and metavirus (**Figure 2**). There was a close relationship between BmERV-47/187 and the insect semotivirus *roo* (**Figure 2**). Surprisingly, class III BmERVs were related to vertebrate retroviruses (**Figure 2**).

### Census of the BmERV Population in the Silkworm Genome

**FIGURE 3:**
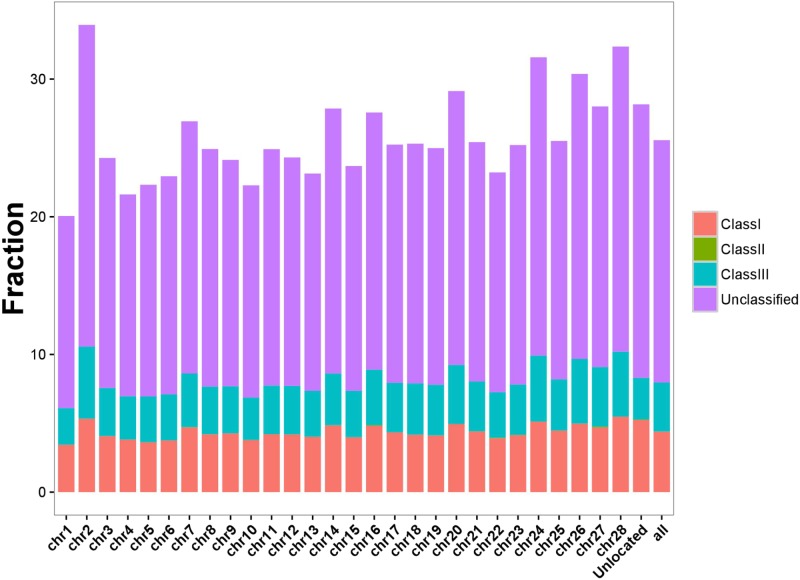
BmERV abundance in different chromosomes. Different BmERV classes are labeled with different colors. The abundance is illustrated using the percentage of the chromosome sequences. “Unlocated” represents BmERV sequences that are not located on a particular chromosome. “All” indicates the percentage of the whole silkworm genome occupied by BmERVs.

To fully assess the abundance of BmERV-derived sequences, RepeatMasker was employed to annotate the silkworm genome using all of the BmERV and non-redundant ERV sequences deposited in Repbase. The BmERV-related sequences annotated by RepeatMasker occupied 25.56% of the silkworm genome (**Supplementary Data Sheet S3**). BmERVs were relatively evenly distributed across the individual chromosomes (**Figure 3**). Chromosome 2 possesses the most BmERVs (**Figure 3**).

### Insertion Time of BmERVs in the Silkworm Genome

**FIGURE 4:**
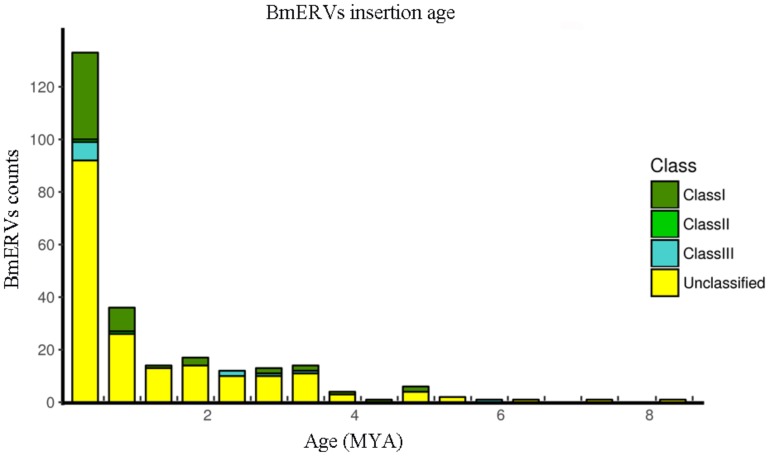
Insertion time of BmERVs. The numbers of different BmERV classes are shown in different colors. Insertion time was estimated by LTR pair divergence. Abbreviation: MYA, million years ago.

As the neutral substitution rate of *B. mori* is still unknown, we applied a rate of neutral substitution in fruitfly that has been previously used for estimating the origination time of silkworm transposable elements (Xia et al., 2004) to date the integration of the BmERVs. Our results showed that BmERVs integrated relatively recently, estimated at less than 10 million years ago (MYA) (**Figure 4**), according to published standards (Garcia-Etxebarria and Jugo, 2016): young ERVs (up to 10–22 MYA), middle-aged ERVs (between 10–22 MYA and 20–43 MYA), and ancient ERVs (more than 43 MYA). The insertion time of most BmERVs was less than 4 MYA (**Figure 4**). Indeed, BmERV-255 was the oldest among the BmERVs, but only inserted about 8.23 MYA (**Supplementary Data Sheet S4**). In addition, 56 of the BmERVs had strictly identical LTR pairs, indicating that these BmERVs inserted very recently (**Supplementary Data Sheet S4**). These data suggest that ERV integration events in *B. mori* occurred recently.

### Comparison of BmERV *env* Genes With F and *GP64* Genes From NPVs

**FIGURE 5:**
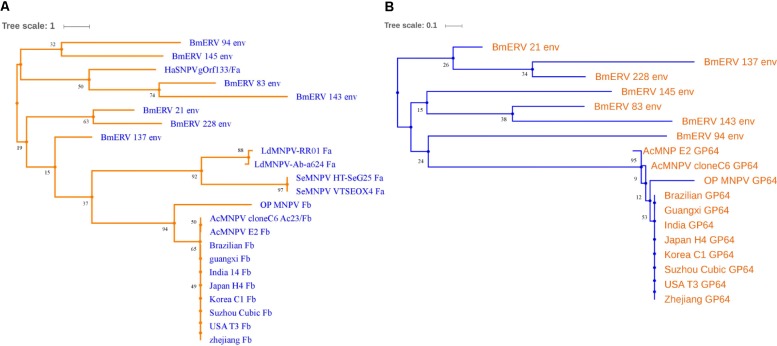
Phylogenetic analysis based on the *env* genes of BmERVs and the F and GP64 genes from NPVs. Group I NPVs including AcMNPV, OpMNPV, and BmNPVs (strains Brazilian, Guangxi, etc.) possess *GP64* and the homologs of F (Fb). Group II NPVs have F (Fa) but lack *GP64*. **(A)** Phylogenetic analysis of the *env* gene of BmERVs and F genes from Group I NPVs (Fb) and Group II NPVs (Fa). **(B)** Phylogenetic analysis of the *env* gene of BmERVs and *GP64* of Group I NPVs. About 500 bp of the *env*-fragments were aligned to generate the phylogenetic tree. AcMNPV, *Autographa californica* nuclear polyhedrosis virus; OpMNPV, *Orgyia pseudotsugata* multinucleocapsid nuclear polyhedrosis virus; BmNPV, *Bombyx mori* nuclear polyhedrosis virus; HaSNPV, *Helicoverpa armigera* nuclear polyhedrosis virus; LdMNPV, *Lymantria dispar* multiple nucleopolyhedrovirus; SeMNPV, *Spodoptera exigua* multiple nucleopolyhedrovirus.

To explore the relationship between *env* gene of BmERVs and genes encoding the envelope protein (Env) in NPVs, we analyzed the evolutionary relationships and homology among these genes. Fa and Fb represent the F protein of Group II NPVs and the homologs of the F protein from Group I NPVs, respectively. The results showed that the *env* sequences of BmERVs were distantly related to genes encoding F and GP64 proteins from Group I and Group II NPVs (**Figures 5A,B**). Surprisingly, one of the phylogenetic lineages contained BmERVs *env* genes and HaSNPVgOrf133 (**Figure 5A**). Indeed, there were a few regions of the alignment where BmERVs *env* genes were not similar to each other. The BmERV *env* genes shared low homology with the F and *GP64* genes from Group I and Group II NPVs (**Supplementary Figure S2**).

**FIGURE 6:**
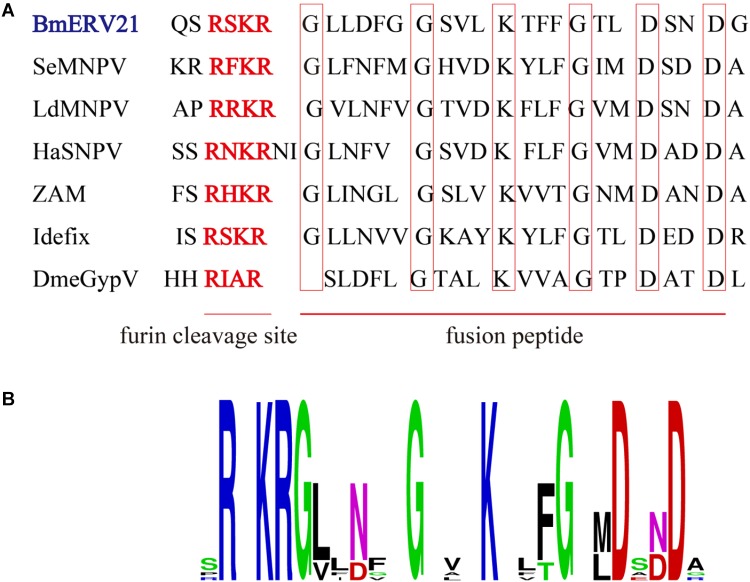
Conserved amino acid sequence block in the Env protein of BmERVs and IERVs and Group II NPV F proteins. **(A)** Multiple alignment of the conserved amino acid sequence block showed a consensus furin cleavage site RxxR and the peptide fusion consensus sequence in the Env protein of BmERV-21, ZAM, Idefix, DmeGypV, and the F protein of SeMNPV, LdMNPV, and HaSNPV. **(B)** Logo visualization of the furin cleavage site and peptide fusion consensus sequence performed using WEBLOGO (weblogo.berkeley.edu/logo.cgi).

Interestingly, we found that the amino acid sequences of BmERV-21 shared a region of similarity with the fusion protein of Group II NPVs and known insect ERVs, which included the furin cleavage signal and downstream predicted fusion peptide (**Figure 6A**). Logo representation of the furin-like consensus motif (RxxR) and peptide fusion consensus sequences (GxxxxxGxxxKxxxGxxDxxD) between BmERV-21 and *Idefix*, *ZAM*, LdMNPV, and SeMNPV are shown in **Figure 6B**, suggesting that the *env* gene of BmERV-21 might encode a fusion protein. However, similar fusion peptide sequences were not found in BmERV-83, BmERV-94, BmERV-137, BmERV-143, BmERV-145, BmERV-228, or the fusion proteins (GP64 and Fb) of BmNPVs.

### Integration and Effective Transcription of the BmERV *env* Genes

**FIGURE 7:**
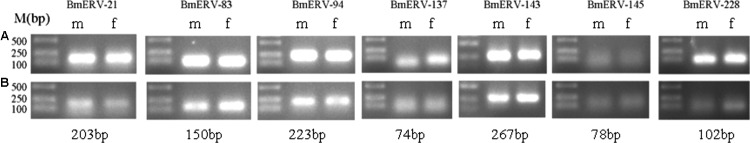
Integration and effective transcription of the BmERV *env* genes in the tissues of *Bombyx mori*. **(A)** PCR with DNA templates was used to verify the presence of *env* genes in the silkworm genome. Tissues samples of DaZao were extracted from midguts (m) and fat bodies (f). **(B)** RT-PCR was employed to detect the expression of the *env* genes of BmERV-21, BmERV-83, BmERV-94, BmERV-137, BmERV-143, BmERV-145, and BmERV-228 using cDNA templates.

To further verify whether BmERVs *env* genes exist in the silkworm genome and are expressed in silkworm tissues, RCR and RT-PCR were employed to detect BmERV *env* genes using DNA and cDNA templates extracted from the midguts and fat bodies of *B. mori*, respectively. DNA samples from both of midguts and fat bodies produced a band of BmERVs provirus *env*; however, BmERV-145 showed a weaker band than other strains (**Figure 7A**). PCR products were used for direct sequencing analysis. The sequencing results also identified the BmERV *env* sequences, in accordance with the sequences identified by bioinformatics (data not shown). The results demonstrated that BmERV *env* genes indeed integrated into the silkworm genome. Furthermore, RT-PCR results showed that the *env* genes of BmERV-83, BmERV-94, and BmERV-143 were expressed abundantly in the midguts and fat bodies (**Figure 7B**). The expression of the *env* genes of BmERV-21 and BmERV-137 was also detected in midguts and fat bodies (**Figure 7B**). However, BmERV-145 and BmERV-228 *env* expression was relatively weaker in midguts and fat bodies (**Figure 7B**). The results suggest that BmERV elements can be effectively transcribed in silkworm tissues.

## Discussion

This study attempted to systematically identify and characterize ERVs in the silkworm genome. By combining two different *de novo* mining strategies, we identified 256 potential BmERVs in the silkworm genome. The silkworm genome is composed of 28 chromosomes with female heterogametic constitution: ZZ for male and ZW for female. The existing silkworm genome data does not include W chromosome sequences (Xia et al., 2014); however, it have been found that many retrotransposon elements have accumulated on the W chromosome of *B. mori* (Abe et al., 2000, 2001, 2005). Thus, more BmERVs will likely be identified when *B. mori* W chromosome sequence data are published.

Phylogenetic analysis based on the RT region was used to cluster BmERVs into nine putative families and three groups. As expected, we found most of the BmERVs were related to IERVs such as *DmeZamV* and *DmeGypV*, according to the phylogenetic analysis. However, BmERVs of class III were related to vertebrate retroviruses. In *D. melanogaster*, phylogenetic analysis shows that three kinds of retrotransposons are similar to class I, II, and III retroviruses, respectively (Llorens C. et al., 2008). LTR retrotransposons or retroviruses in insects and vertebrate retroviruses have a common evolutionary history and should be considered in parallel (Nefedova and Kim, 2017).

Due to using a substitution rate of *Drosophila*, and the homogenization of the LTR pairs caused by the effect of recombination or gene conversion events, the insertion time proposed for BmERVs is only an estimate. Similarly, the rate of neutral substitution in fruitfly has been previously used for estimating the origination time of silkworm transposable elements (Xia et al., 2004). The oldest bovine ERV was inserted between 126 and 58 MYA (Garcia-Etxebarria and Jugo, 2010). The insertion time of ERV elements in humans and in chimpanzees is 19.5–8.9 MYA and 33–15.8 MYA, respectively (Garcia-Etxebarria and Jugo, 2010). However, our data show that all of the BmERVs are young members, with the oldest insertion dated to approximately 8 MYA. Thus, BmERVs inserted into the silkworm genome recently.

In addition, the proportion of BmERVs in the genome was also different from that in vertebrates. ERVs often occupy a substantial fraction of genomes, accounting for about 5% of bat, 10% of mouse, and 8% of human genome sequences (Zhuo et al., 2013). In this work, using a rigorous set of criteria, we estimated that BmERVs occupied ∼25% of the silkworm genome, which is significantly higher than the proportion identified in vertebrates. IERVs have been mostly reported in *Drosophila*, but to our knowledge their quantity in genome-wide is still unknown. The repeat elements in the silkworm genome represent approximately 43.6%, which is significantly higher than that detected in other insect species including 12 *Drosophila* species (Osanai-Futahashi et al., 2008; Xia et al., 2014). We speculated that ERV elements in *B. mori* would also represent a larger proportion of the genome than those in *Drosophila*. Nevertheless, in this study, no full-length BmERVs were identified in the silkworm genome. On the contrary, in other insects, several ERVs have been identified, and most of them possess a complete retroviral structure (Pelisson et al., 2002; Akkouche et al., 2012). Thus far, only the well-known *gypsy* and *ZAM* elements of *D. melanogaster* have been shown to possess infectious properties (Leblanc et al., 2000; Llorens J.V. et al., 2008). In general, the infectious ability of IERVs is thought to be associated with the expression of Env encoded by the *env* gene (Teysset et al., 1998).

However, only seven BmERVs possessing *env* genes were identified in the silkworm genome. Furthermore, according to the sequences identified by bioinformatics, we found that the BmERV *env* genes did not contain a full-length ORF. It is believed that some LTR retrotransposons which lacked the env gene integrated into the dsDNA genome of a baculovirus and “captured” env gene from baculoviruses (Malik et al., 2000). Two different envelope proteins, named GP64 and F, have been identified in the family *Baculoviridae* (Pearson and Rohrmann, 2002). In general, Group I baculoviruses possess GP64 and the homologs of the F protein (Fb), whereas Group II baculoviruses have the F protein (Fa) but lack GP64 (Pearson and Rohrmann, 2002). Indeed, IERV Env proteins showed significant homology to the fusion protein FP of Group II baculoviruses and also have fusogenic properties (Rohrmann and Karplus, 2001; Misseri et al., 2003, 2004). However, only the BmERV-21 amino acid sequence shared a similar putative furin-like cleavage site and fusion peptide with Group II baculoviruses. Further experimental detection is needed to confirm whether the sequences of BmERV-21 and its *env* gene are complete. To date, no complete BmERV sequence has been verified in living tissues or cell lines, not to mention the functional research. In addition, except for HaSNPVgOrf133, we found that the *env* sequences of BmERVs were distantly related to genes encoding F (Fa and Fb) and GP64 protein from Group I and Group II NPVs. Thus, BmERVs might have “captured” an *env* gene from HaSNPV, and this event would have happened a long time ago. We speculated that *B. mori* might have been infected by various baculoviruses before domestication. But for the moment, *B. mori* does not seem to be infected with HaSNPV. Compared to wild insects, *B. mori* is a completely domesticated insect with a good living environment and, as such, could be largely exempt from long-term baculovirus infection. Only healthy individuals are used to produce and reproduce. Moreover, we estimated that ERV integration events in *B. mori* occurred recently. Therefore, we speculate that the domestication and recent integration events might account for only seven *env* genes being detected in the silkworm genome. If the ERVs of wild silkworm were identified and compared with those of BmERVs, a greater understanding of the evolution of BmERVs would be achieved.

ERVs occupy a significant portion of the host genome. The functions of ERVs are of particular interest, serving as a regulatory factor to influence host gene expression (Suntsova et al., 2015). Additionally, functional ERV genes such as *env* encode active proteins, which may exert important physiological functions in the host (Suntsova et al., 2015). Our results confirmed that the BmERVs *env* genes existed in the silkworm genome and exhibited transcriptional activity in the midguts and fat bodies. Thus, BmERV *env* genes might also play an important role in host biology, especially in antiviral defense. In fact, there have been calls for attention to the role of IERVs in the immune response (Fablet, 2014).

Taken together, this is indeed the first genome-wide approach for the screening and characterization of BmERVs. Further studies are thus needed to verify whether the BmERVs possess complete structures and whether infectious properties exist *in vivo*. Such studies would elucidate the roles of BmERVs in *B. mori*.

## Author Contributions

MF participated in the design of the study and drafted the manuscript. MF and XW collected and analyzed the data. FR helped in the sample collection. NZ and YZ performed PCR and RT-PCR. JS participated in the design and coordination of the study, and drafted the manuscript. All authors read and approved the final manuscript.

## Conflict of Interest Statement

The authors declare that the research was conducted in the absence of any commercial or financial relationships that could be construed as a potential conflict of interest.

## Footnotes

1 http://silkworm.genomics.org.cn/

2 http://www.gydb.org/index.php/Main_Page

3 http://www.repeatmasker.org/

4 http://weblogo.berkeley.edu/logo.cgi

## Supplementary Material

FIGURE S1 Structure of 256 BmERVs.

FIGURE S2 Homology comparison between *env* of BmERVs and the F and *GP64* genes of NPVs. **(A)** Homology of *env* of BmERVs and F genes from Group I NPVs (Fb) and Group II NPVs (Fa). **(B)** Homology of *env* of BmERVs and *GP64* of Group I NPVs.

TABLE S1 Reverse transcriptase (RT) domain from vertebrate retroviruses and insect LTR- retrotransposons used in this study.

TABLE S2 Primers used for amplifying the env genes of BmERV.

DATA SHEET S1 Details of all of BmERVs.

DATA SHEET S2 Classification of BmERVs.

DATA SHEET S3 Census of BmERVs in the silkworm chromosome.

DATA SHEET S4 Insertion time of BmERVs in the silkworm genome.
