# Supplementary figures and images for: Genome-Wide Characterization of Endogenous Retroviruses in Bombyx mori Reveals the Relatives and Activity of env Genes
Source: Front Microbiol. 2018 Aug 3;9:1732. doi: 10.3389/fmicb.2018.01732 (PMC6085415; doi:10.3389/fmicb.2018.01732)

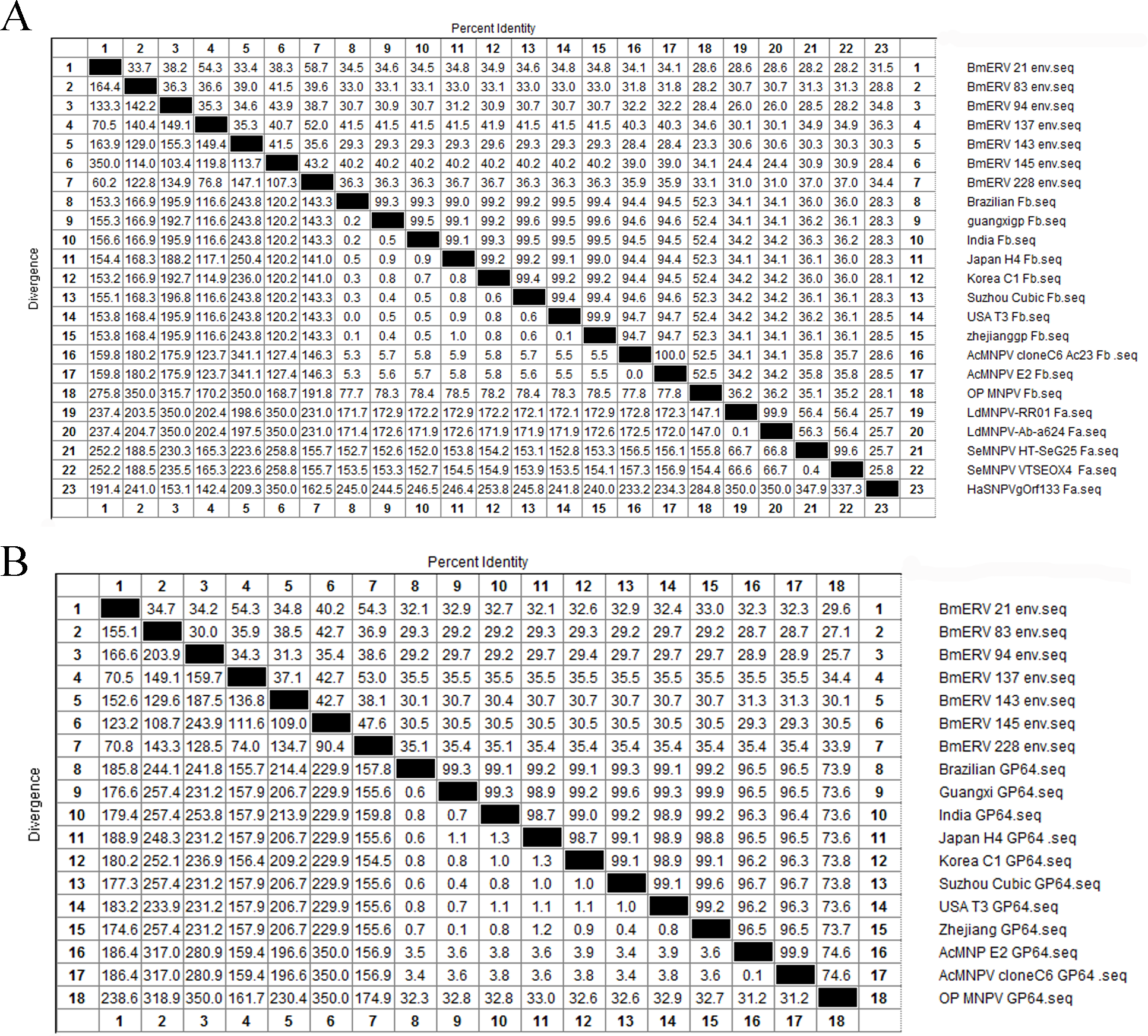

Supplement: FIGURE S2 — Homology comparison between env of BmERVs and the F and GP64 genes of NPVs. (A) Homology of env of BmERVs and F genes from Group I NPVs (Fb) and Group II NPVs (Fa). (B) Homology of env of BmERVs and GP64 of Group I NPVs. [file Image_2.TIF]
