# Supplementary material for: Genome-Wide Characterization of Endogenous Retroviruses in Bombyx mori Reveals the Relatives and Activity of env Genes
Source: Front Microbiol. 2018 Aug 3;9:1732. doi: 10.3389/fmicb.2018.01732 (PMC6085415; doi:10.3389/fmicb.2018.01732)
Supplement: TABLE S1 — Reverse transcriptase (RT) domain from vertebrate retroviruses and insect LTR- retrotransposons used in this study. [file Table_1.docx]

**Supplementary Table S1. The reverse transcriptase (RT) domain from vertebrate retrovirus and insecte LTR- retrotransposon used in this study.**

| Abbreviation | Species Name | Accession number |
| --- | --- | --- |
| Vertebrate retrovirus |  |  |
| HERV-W | Multiple sclerosis associated retrovirus | AAB66528.1 |
| RERV | rabbit endogenous retrovirus | AAM81191.1 |
| GH-G18 | Golden hamster intracisternal  A-particle H18 | P04026.1 |
| MMTV | mouse mammary tumor virus | AAG18012.1 |
| ALV | Avian leucosis virus | NP_955611.1 |
| Python-molurus | Python molurus endogenous retrovirus | AAN77283.1 |
| HML9 | human MMTV-like 9 | AAB63113.1 |
| HML6 | human MMTV-like 6 | AAC14144.1 |
| SnRV | Snakehead retrovirus | AAC54861.1 |
| WDSV | Walleye dermal sarcoma virus | AAC82611.1 |
| SRV-1 | Simian retrovirus 1 | AAA47732.1 |
| JSRV | Jaagsiekte sheep retrovirus | AAA89182.1 |
| HERV-Fc2 | Human Endogenous Retrovirus Fc2 | XP_012299082.1 |
| HERV-Fc1 | Human Endogenous Retrovirus Fc1 | XP_013971778.1 |
| HFV | human foamy virus | AAA66556.1 |
| HERV-L | Human endogenous retrovirus with leucine tRNA primer | CCD13217.1 |
| FeFV | Feline foamy virus | CAA11581.1 |
| HIV-1 | Human immunodeficiency virus 1 | AGF41802.1 |
| BLV | bovine leukemia virus | P03361.1 |
| BaEV | Baboon endogenous virus | YP_009109694.1 |
| FELV | feline leukemia virus | NP_955579.1 |
| MuLV | murine leukemia virus | 1I6J_A |
| PERV | porcine endogenous retrovirus | AAC16767.1 |
| MDEV | Mus dunnie ndogenous virus | AAC31805.1 |
| GALV | gibbon ape leukemia virus | YP_002000606.1 |
| KoRV | koala retrovirus | AHY24803.1 |
| Insect errantivirus |  |  |
| DmeGypV | Drosophila melanogaster Gypsy virus | M12927 |
| Dwe176V | Drosophila melanogaster 17-6 virus | X01472 |
| Dme297V | Drosophila melanogaster 297 virus | X03431 |
| DmeZamV | Drosophila melanogaster ZAM virus | AJ000387 |
| DmeIdeV | Drosophila melanogaster Idefix virus | AJ009736 |
| DmeNomV | Drosophila melanogaster nomad virus | AF039416 |
| DmeTirV | Drosophila melanogaster tirant virus | X93507 |
| Dan Tom V | Drosophila ananassae tom virus | Z24451 |
| DsuGypV | Drosophila subobscura Gypsy virus | X72390 |
| DviGypV | Drosophila virilis Gypsy virus | M38438 |
| DviTvIV | Drosophila virilis Tv1 virus | AF056940 |
| TniTedV | Trichoplusia ni TED virus | M32662 |
| CcaYoyV | Ceratitis capitata yoyo virus | U60529 |
| Insect metavirus |  |  |
| blastopia | Drosophila melanogaster blastopia | Z27119.1 |
| micropia-DM11 | Drosophila melanogaster micropia-Dm11 retrotransposon | X14037.1 |
| mdg3 | D.melanogaster mdg3 retrotransposon | X95908.1 |
| mdg1 | D.melanogaster mgd1 retrotransposon | X59545.1 |
| Insect semotivirus |  |  |
| roo | Drosophila melanogaster roo transposon | AY180917.1 |
