# Supplementary material for: Genome-Wide Characterization of Endogenous Retroviruses in Bombyx mori Reveals the Relatives and Activity of env Genes
Source: Front Microbiol. 2018 Aug 3;9:1732. doi: 10.3389/fmicb.2018.01732 (PMC6085415; doi:10.3389/fmicb.2018.01732)
Supplement: TABLE S2 — Primers used for amplifying the env genes of BmERV. [file Table_2.DOCX]

**Supplementary Table S2. Primers used for amplifying the *env* genes of BmERV**

| **Target** | **Primer** | **Sequence(5’-3’)** | **Length(bp)** |
| --- | --- | --- | --- |
| **BmERV-21** | Forward | TTGCACCCGACTGTACTCAG |  |
|  | Reverse | ACTTGAGGTAACACGAGGGG | 203 |
| **BmERV-83** | Forward | AAAATAGCCCCGCGTTGTGA |  |
|  | Reverse | TATGACTGTCGTCGCGAAACC | 150 |
| **BmERV-94** | Forward | ACGCGTTCCTAAGTCTGACA |  |
|  | Reverse | TCGTCTTCATCCATGGTGCC | 223 |
| **BmERV-137** | Forward | TCACGTGTTAAACGAGAATTGTTTG |  |
|  | Reverse | TGTGCATCAGGTGTACCGATTA | 74 |
| **BmERV-143** | Forward | TTGAATCCCGACACGAGGTC |  |
|  | Reverse | CGGTTGACTCTACAAGCTCCA | 267 |
| **BmERV-145** | Forward | CTTACACAAAATTACC |  |
|  | Reverse | GATTGTGAGGCACCTGTA | 78 |
| **BmERV-228** | Forward | TGCACAAGGTCAAACGCTCT |  |
|  | Reverse | CGATCGCAGCGTCGTATTTC | 102 |
